# A Novel Insecticidal Spider Peptide that Affects the Mammalian Voltage-Gated Ion Channel hKv1.5

**DOI:** 10.3389/fphar.2020.563858

**Published:** 2021-01-13

**Authors:** Diana Alvarado, Samuel Cardoso-Arenas, Ligia-Luz Corrales-García, Herlinda Clement, Iván Arenas, Pavel Andrei Montero-Dominguez, Timoteo Olamendi-Portugal, Fernando Zamudio, Agota Csoti, Jesús Borrego, Gyorgy Panyi, Ferenc Papp, Gerardo Corzo

**Affiliations:** ^1^ Departamento de Medicina Molecular y Bioprocesos, Instituto de Biotecnología, Universidad Nacional Autónoma de México, Cuernavaca, México; ^2^ Departamento de Alimentos, Facultad de Ciencias Farmacéuticas y Alimentarias, Universidad de Antioquia, Medellín, Colombia; ^3^ Department of Biophysics and Cell Biology, Faculty of Medicine, University of Debrecen, Debrecen, Hungary

**Keywords:** atrial fibrillation, Kv1.5, *Oculicosa supermirabilis*, recombinant expression, spider venom

## Abstract

Spider venoms include various peptide toxins that modify the ion currents, mainly of excitable insect cells. Consequently, scientific research on spider venoms has revealed a broad range of peptide toxins with different pharmacological properties, even for mammal species. In this work, thirty animal venoms were screened against hK_v_1.5, a potential target for atrial fibrillation therapy. The whole venom of the spider *Oculicosa supermirabilis*, which is also insecticidal to house crickets, caused voltage-gated potassium ion channel modulation in hK_v_1.5. Therefore, a peptide from the spider *O. supermirabilis* venom, named Osu1, was identified through HPLC reverse-phase fractionation. Osu1 displayed similar biological properties as the whole venom; so, the primary sequence of Osu1 was elucidated by both of N-terminal degradation and endoproteolytic cleavage. Based on its primary structure, a gene that codifies for Osu1 was constructed *de novo* from protein to DNA by reverse translation. A recombinant Osu1 was expressed using a pQE30 vector inside the *E. coli* SHuffle expression system. recombinant Osu1 had voltage-gated potassium ion channel modulation of human hK_v_1.5, and it was also as insecticidal as the native toxin. Due to its novel primary structure, and hypothesized disulfide pairing motif, Osu1 may represent a new family of spider toxins.

## Introduction

Spider venoms are a heterogeneous mixture of molecules that range from enzymes to toxic peptides and small organic components (Pineda et al., 2014). Among the toxic peptides, there are disulfide-rich neurotoxins harmful to insects, and perhaps due to molecular serendipity, some of them are also toxic to mammals, which affect cell receptors, especially ion channels. So, most of the spider neurotoxins could be considered, from the molecular perspective, as precious and unique molecules to help us to understand some of the ion channels’ mechanisms that are important for physiological purposes (Nicholson and Graudins, 2002). Also, it is well known that spider peptide toxins tend to be promiscuous concerning their selectivity for ion channels. Still, some could be specific and exclusive to unveil relevant domains of the ion channel structures (Corzo and Escoubas, 2003). For example, the spider δ-atracotoxins (δ-ACTXs), that belong to the NaSpTx spider family 4, are disulfide-rich neurotoxins that modify the voltage-gated sodium channels (Na_v_), both in insects and in mammals. Those neurotoxins bind to domain IV in the S3/S4 loop, also identified as neurotoxin receptor site-3 (Clairfeuille et al., 2019), decreasing the fast inactivation of Na_v_s (Gunning et al., 2003). Since δ-ACTXs have this dual effect on both mammals and insects, they have facilitated our understanding of some of the molecular interactions with Na_v_ (Borrego et al., 2020). Thus, the search for new spider peptide structures that could contribute to ion channel physiology knowledge should be granted and embraced. In this tenor, the advent of transcriptomics and proteomics for studying spider venom glands and venoms, respectively, have exponentially uncovered a significant number of primary structures from spider venoms (Zhang et al., 2010; Quintero-Hernandez et al., 2011; Jiang et al., 2013; Oldrati et al., 2017; Langenegger et al., 2019). Nevertheless, understanding the mechanism of action of most spider structures already found has been hampered mainly because of insufficient infrastructure in both material and academic to obtain enough quantities of such spider peptides by natural, synthetic, or recombinant means (Quintero-Hernandez et al., 2011). Also, it has been limited because of the low capacity of most research labs to test a wide range of a growing number of ion channels and their subtypes compared to the known motifs in spiders to discern their correct cellular or molecular targets.

In this work, we look for spider peptides that target the voltage-gated potassium channels (K_v_), specifically the hK_v_1.5 potassium channel, in which ion currents are also referred to IKur, and they are the main ion currents in the repolarization of the atrial action potential (AP). IKur has been observed in human atrial myocytes, but it is absent in the human ventricle (Fedida et al., 1993). Several researchers have concluded that the blockage of IKur could prolong the duration of AP of atrial fibrillation (AF) in patients (Brunner et al., 2003; Guo et al., 2016), and stop fibrillation, indicating that hK_v_1.5 is potentially a selective target and safe strategy for AF therapy (Lip and Tse, 2007; Ehrlich et al., 2008; Ford and Milnes, 2008; Ehrlich and Nattel, 2009; Ravens, 2010). Therefore, one of our research goals is to discover selective hK_v_1.5 peptide inhibitors and test them in an AF model. At present, all of the known hK_v_1.5 blockers are small molecules (Gutman et al., 2005; Wettwer and Terlau, 2014; Bajaj and Han, 2019). However, they are usually not as selective as large molecules, e.g., peptides, which have a much larger interacting surface with ion channels than the small molecules, and the larger the interaction, the higher the selectivity and the lower the risk of side effects (Corzo et al., 2008; Ali et al., 2019). Perhaps one of the main reasons for the absence of hK_v_1.5 peptide inhibitors is because this subtype of ion channel does have a positively charged Arg residue in its selectivity pore, unlike other K_v_ ion channels (Zhu et al., 2005), which prevents the potential blocking peptides from binding to hK_v_ channels. So, here we report the primary structure of a spider toxin, named Osu1, that affects hK_v_1.5, and it seems to be one of the first electric-current modifier peptides of this ion channel. One of the peculiarities of Osu1, according to our results, is that it does not bind to the selectivity pore, but the voltage-sensing domain; so, it does not block the pore of hK_v_1.5 but prevents its opening at physiological membrane potentials. Nevertheless, the result would be the same; if there is no K^+^ ion flow through hK_v_1.5, i.e., no IKur current, and if there is no repolarizing outward IKur current during the atrial AP of an AF patient, the duration of AP would be prolonged, and fibrillation will be abolished (Guo et al., 2016). Besides the effects of Osu1 on hK_v_1.5, it is also insecticidal, and according to its amino acid sequence, it could be placed in a new spider toxin family; that is, none of the already proposed spider toxin families (Klint et al., 2012) has a primary structure similar to Osu1. Furthermore, most of the spider peptides with significant identity (90–95%) to Osu1 have been found just as transcripts, and only a spider peptide toxin with 48% identity to Osu1, ω-agatoxin-IA, has biochemical properties already reported (Santos et al., 1992). Finally, based on software that predicts three-dimensional protein structures, Osu1 may have a different disulfide-pairing motif than the known spider peptides.

## Material and Methods

### Strains, Vectors, and Enzymes

Bacterial strains: *E. coli* XL1-Blue (cloning) (*gyrA96 recA1 hsdR17 endA1 thi-1 relA1 supE44 lac* [F′ *proAB* Tn*10* (Tetr) *lacI* ^q^ZΔ*M15*]) (Agilent, United States); and *E. coli* SHuffle^®^T7 (Expression) (*pro*, F´ *lac*, *lacI* ^*q*^ araD139 Δ(ara-leu)7697 lacZ::T7 fhuA2 gene1 Δ(phoA)PvuII ahpC* phoR galE (or U) ΔtrxB rpsL150(Str^R^ *galK λatt*:pNEB3-r1-*cDsbC* (Spec^R^, *lacI* ^*q*^)) *Δ(malF)3 Δgor*) (New England BioLabs, Ipswich, MA, United States), respectively. Plasmid pQE30 (Qiagen, CA, United States) was used for cloning the Osu1 gene, and production of the 6His-tagged recombinant Osu1 (rOsu1). The enzymes were from New England Biolabs (NEB, Ipswich, MA, United States) (Taq-Polymerases, Vent-Polymerase, restriction enzymes), T4 Ligase from Fermentas (Carlsbad, CA, United States).

### Isolation and Chemical Characterization of Osu1

The venom from the spider *Oculicosa supermirabilis* was extracted by electrical stimulation. The spiders were field-collected in the Kazakhstan Republic (Fauna Ltd.). At the Institute of Zoology in Almaty, Kazakhstan, the spiders were identified. This species is found in Kazakhstan, Uzbekistan, and Turkmenistan (Logunov and Gromov, 2011). The raw venom (the venom of more than 200 individuals, females, and males were milked and pooled, to yield 2 mg) was dissolved in water with 0.1% of trifluoracetic acid (TFA) and then centrifuged to remove all the insoluble material (14,000 *g* for 5 min). The liquid phase was injected directly for fractioning using High-Performance Liquid Chromatography (HPLC). The venom mixture was separated using a reverse-phase analytical C_18_ column (5C_18_MS, 4.6 × 250 mm, Vydac, United States) equilibrated in 0.1% TFA, and eluted with 0–60% acetonitrile in 0.1% TFA in a linear gradient, run for 60 min (1 ml/min) (Corzo et al., 2001). The elution fractions were monitored at 280 nm, collected in 1.5 ml vials, and dehydrated under a high vacuum. The dried samples were used first to conduct electrophysiological assays. Those fractions capable of affecting hK_v_1.5 channels were subjected to another purification process using a C_18_ reverse-phase column (4.6 × 250 mm, Vydac, United States) equilibrated in 0.1% TFA, and eluted with 20–60% acetonitrile in 0.1%TFA in a linear gradient, and run for 40 min (1 ml/min). The new fractionated components were again subjected to electrophysiological assays. It was analyzed by mass spectrometry using a Thermo Scientific LCQ Fleet ion trap mass spectrometer (San Jose, CA, United States) with a Surveyor MS syringe pump delivery system. The pure peptide was also subjected to Edman degradation using an LF3000 Protein Sequencer (Beckman, CA, United States), and endoproteolytic digestions to determine its primary structure, as reported previously by our group (Corzo et al., 2008).

### Osu1 Gene Construction

• The primary structure of the peptide Osu1 was used to do a reverse translation and thus generate a DNA sequence (https://www.bioinformatics.org/sms2/rev_trans.html). Afterward, the obtained sequence was analyzed and adjusted, complying with the preferential codon usage of *E. coli* (http://www.kazusa.or.jp/codon). Then, we designed four overlapping synthetic oligonucleotides (Supplementary Table S1) to construct the Osu1 gene. Additionally, the recognition strings for *Bam*HI (GGATCC) and Factor Xa protease (ATCGAGGGAAGG) were added at the beginning of oligonucleotide Osu1-Up1. Two stop codons (TAATAG) and the restriction sequence for *Pst*I (CTGCAG) were added to the end of oligonucleotide Osu1-Lw4.
• The Osu1 gene was constructed *in vitro* using the “overlapping oligonucleotide extension” following the Polymerase Chain Reaction (PCR). In a few words, Osu1-Lw2 plus Osu1-Up3 oligonucleotides (17 bp overlap) were mixed in 0.1 pmol/µl final concentration each, with the other components in the reaction mixture including Vent polymerase for PCR, and then amplified in eight cycles under the following conditions: 94°C/30 s, 58°C/30 s, and 72°C/30 s. After the eighth cycle, oligonucleotides Osu1-Up1 plus Osu1-Lw4 were added to the reaction mixture (0.4 pmol/µl final concentration each) and followed by 25 amplification cycles with the following conditions: 94°C/30 s, 60°C/40 s, and 72°C/30 s. A final elongation step was carried out at 72°C/10 min. The PCR product was run on 1% agarose gels containing GelRed^®^ (Biotium, Fremont, CA, United States) and envisioned under ultraviolet (UV) light (DNA marker from NEB, Ipswich, MA, United States). Afterward, the amplification product was purified from the agarose gel with the High Pure Plasmid Isolation kit (Roche, Basel, Switzerland).

The assembled and purified gene was digested with *Bam*HI and *Pst*I enzymes (NEB). The gen was run and extracted from agarose gel, then ligated (T4 ligase, Fermentas) to the pQE30 expression plasmid, previously restricted by the same enzymes. The new recombinant plasmid (pQE30/Osu1) was used to transform *E. coli* XL1-Blue cells by heat shock. The plasmid’s antibiotic selection system enabled us to pick some colonies to be tested using PCR (pQE-Fwd (5ʹ-GAG​CGG​ATA​ACA​ATT​ATA​A-3ʹ) and pQE-Rev (5ʹ-GGT​CAT​TAC​TGG​ATC​TAT-3ʹ). Four colonies with the predicted amplification band were subjected to plasmid purification and then sequenced at the Institute of Biotechnology, UNAM, Mexico.

### Expression Screening

The recombinant plasmid pQE30/Osu1, sequence confirmed, was used to transform *E. coli* SHuffle cells for testing expression. Briefly, the transformation procedure was as follows: 50 ng of pQE30/Osu1 plasmid was blended with 100 µl of competent *E.* coli/SHuffle cells and maintained in ice for 30 min, then heated the mix for 1 min at 42°C, followed by cooling in ice for 5 min. Afterward, we added 220 µl of Super Optimal broth with Catabolite repression (SOC) medium, and the mix was kept for 60 min at 37°C. After incubation, 50 µl of the mixture were spread over Petri dishes containing (Luria-Bertani) LB agar-media with ampicillin (100 µg/ml) (Sigma, St. Louis, MO, United States). Grown colonies that harbored the pQE30/Osu1 vector were used to screen their expression. Colonies individually were selected, and seeding each in 3 ml of LB broth, including ampicillin plus 1 mM isopropyl ß-D-thiogalactoside (IPTG, Sigma, St. Louis, MO, United States). Then they were incubated overnight in a shaker at 250 rpm and 37°C. Expression was tested qualitatively by SDS-PAGE. Lastly, a positive clone was selected to evaluate the expression of Osu1.

### Expression of Recombinant Osu1

rOsu1 was produced in the *E. coli* SHuffle strain. LB broth was used to cultivate cells until an optical density (*OD*_*600*_) of 0.6. At that point, 0.5 mM of IPTG was added to induce the peptide expression. Induced cells were maintained for 8 h at 25°C and then collected by centrifugation (5,500 *g*, 20 min, 4°C). Using a mechanical system (One-Shot Cell Disruptor from Constant Systems, Northants, United Kingdom), the cells were burst down. The disrupted cells were subjected to centrifugation (10,000 *g*, 20 min, 4°C) to separate inclusion bodies, which were solubilized using guanidine hydrochloride (GdHCl) 6M, Tris HCl 50 mM, pH 8. Employing Ni-NTA agarose (Qiagen, CA, United States), we purify the recombinant peptides from the dissolved inclusion bodies. Afterward, the peptide was reduced using dithiothreitol (DTT) (Sigma-Aldrich, Ontario, Canada) for 1 h at 37°C. The product was subjected to analytical RP-HPLC (C_18_ column 4.6 × 250 mm, Vydac, United States) using 20–60% acetonitrile in 0.1% TFA in a linear gradient, and run for 40 min (1 ml/min). The obtained reduced fractions were folded *in vitro*. Briefly, the reduced peptide (50 µg/ml) was added to a refolding buffer (0.1 M Tris, pH 8, 2 M GdHCl, 1 mM GSSG, and 10 mM GSH). The mixture was allowed to oxidize for 4 days at 4°C. After that, it was driven to pH 2 by TFA addition. The folded peptide was cleaned by analytical RP-HPLC (C_18_ column 4.6 × 250 mm, Vydac, United States) using the gradient previously mentioned.

### *In vivo* Biological Activity

Fractions obtained from RP-HPLC were tested in mice (strain CD-1, 17–21 g) by intracranial (ic) injection and in house-crickets (*Acheta domesticus,* 0.1–0.16 g) by lateroventral thoracic injection (lv). Osu1 was not toxic to mice up to 5 µg/mouse. The median paralyzing dose (PD_50_) in crickets was defined as the amount of peptide that produces the paralysis of 50% of the population of crickets experimentally evaluated. The median lethal dose (LD_50_) in crickets was defined as the amount of peptide that produces the death of 50% of the treated population. The PD_50_ and LD_50_ were determined using the Dixon method (Dixon, 1965). In brief, one cricket each time was dosed with established doses within regular periods. If the first cricket was paralyzed at least 1 min within the first 10 min after the inoculation, the next cricket was injected with a lower dose. Similarly, if the first cricket was death after 30 min following the injection, the next cricket was inoculated with a lower dose. This progression proceeded until required insects were dosed for calculating either the PD_50_ or the LD_50_. The mean and the confidence intervals of either the PD_50_ or the LD_50_ were determined, according to Dixon (1965). Experiments with animals were earlier accepted by the Bioethics Committee of the Biotechnology Institute (project No. A1-S-8005) and conducted complying with proper regulations.

### Electrophysiology

Murine erythroleukemia (MEL) cells stably expressing hK_v_1.5 channels were maintained following usual conditions, as described before (Grissmer et al., 1994) and were a gift from Dr. Heike Wulff. According to standard protocols, voltage-clamped cells were used to measure the whole-cell currents (Corzo et al., 2008). A Multiclamp 700B amplifier attached to a personal computer (1322A data acquisition hardware, Molecular Devices, Sunnyvale, CA) was employed. A series resistance compensation up to 70% was used to achieve good voltage-clamp conditions and minimize voltage errors. Leitz Fluovert (Leica, Wetzlar, Germany) or Nikon TE2000-U fluorescence microscopes were used to observing cells. Pipettes were pulled from GC 150 F-15 borosilicate glass capillaries Harvard Apparatus (Kent, United Kingdom) in five stages, which resulted in electrodes with 3–5 MOhm resistance in the bath. The composition of the bath solution was 5 mM KCl, 145 mM NaCl, 2.5 mM CaCl_2_, 1 mM MgCl_2_, 10 mM HEPES, 5.5 mM glucose, 0.1 mg/ml bovine serum albumin (Sigma-Aldrich), and, pH 7.35. From a holding potential of −100 mV, voltage steps to +50 mV were applied for ionic current measurements every 15 s. The pClamp10 software package (Molecular Devices) was used for data acquisition and analysis. Whole-cell current traces were adjusted for ohmic leakage, before analysis and were digitally filtered (three-point boxcar smoothing). The current activation kinetics were characterized by implementing a single-exponential function (f(t) = A*Exp(−t/tau)+C).

### Secondary Structure of Recombinant Osu1

The secondary structure of rOsu1 was evaluated by circular dichroism (CD). The measurement was carried out on a Jasco model J-720 spectropolarimeter (Jasco, Tokyo, Japan), from 250 to 190 nm in an aqueous solution of 60% trifluoroethanol (TFE), at room temperature, with a 1-mm pathlength cell. Data were collected at 1 nm with a scan rate of 20 nm/min, and a time constant of 0.5 s. The concentration of rOsu1was 60 μM. Data was the average of three separate recordings and analyzed by the software Bestsel (http://bestsel.elte.hu/index.php) (Micsonai et al., 2018). A recombinant scorpion neurotoxin (rCssII), previously characterized by NMR and CD, and also a three-finger toxin, was used as comparative controls under the same extent conditions.

### Structural Model of Osu1

The amino acid sequence of Osu1 was used to generate a three-dimensional structure through different modeling programs. These programs are based on different 3D structure prediction techniques, such as I-Tasser (Yang and Zhang, 2015), Swiss-Model (Waterhouse et al., 2018), Robetta (Kim et al., 2004) and Modeller (Webb and Sali, 2016). For Modeller, a sequence alignment between Osu1 and the template, OtTx1a (PDB ID: 2n86), was calculated to guide the modeling, using the T-Coffee homology extension (PSI-coffee) algorithm (Di Tommaso et al., 2011). The NMR structure of the spider toxin OtTx1a (PDB ID: 2n86) was used as a template according to the best parameters found by LOMETS (Local Meta-Threading Server) (Wu and Zhang, 2007). A total of 10,000 models were generated by Modeller, selecting the most representative model using the root mean square deviation (RMSD), DOPE score, and main chain quality through PROCHECK (Dunbrack, 2004). From the set of structures generated by I-Tasser, Swiss-Model, and Robetta, only the models with full Cys oxidation were selected for the final analysis. The final figures were prepared with VMD (Humphrey et al., 1996; Dunbrack, 2004) and ESPrit3.0 (Gouet et al., 1999).

### Statistical Analysis

The SPSS statistical software was used for statistical analysis (SPSS Inc., Chicago, IL, United States). The mean ± standard error of the mean (SEM) and 95% confidence intervals were used to express the data. Student paired *t-test*, analysis of variance (ANOVA), or Tukey’s test (for multiple comparisons) were used to determine the statistical significance. *p* < 0.05 was considered significant.

## Results

### Toxin Purification and Sequencing

**FIGURE 1:**
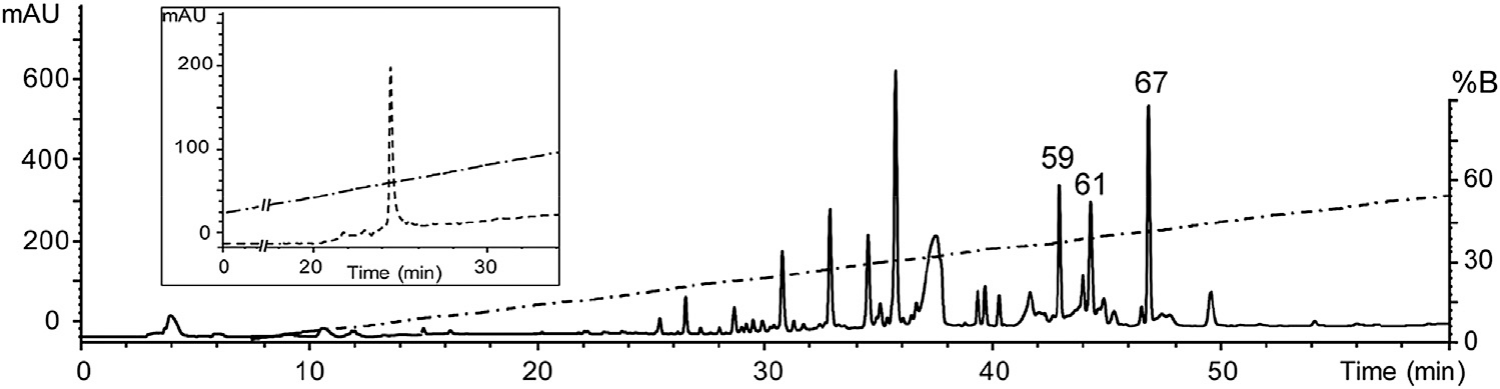
Reverse-phase HPLC chromatogram of the venom of *O. supermirabilis.* The soluble venom of *O. supermirabilis* (obtained from 2 mg crude soluble venom) was fractionated using a reverse-phase analytical C_18_ column (5C_18_MS, 4.6 × 250 mm) equilibrated in 0.1% TFA, and eluted with a linear gradient of acetonitrile (solution B) starting after 5 min from 0 to 60% in 0.1% TFA, run for 60 min at a flow rate of 1 ml/min. The fraction peak #59 in the figure was the fraction that gave positive results in modifying currents in hK_v_1.5, and was further purified to homogeneity (Inside figure) starting after 5 min from 20 to 60% CH_3_CN during 40 min at a flow rate of 1 ml/min.

**FIGURE 2:**
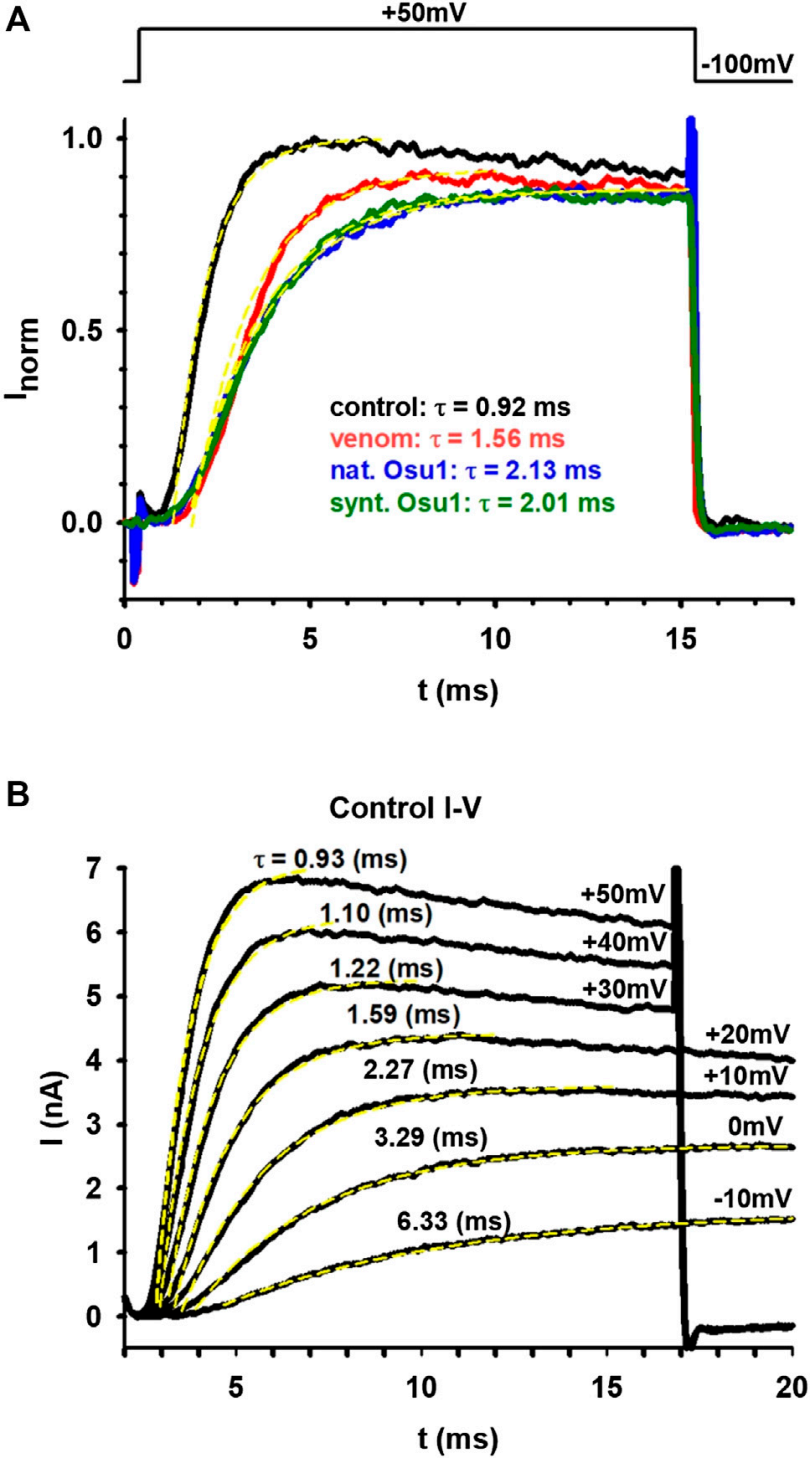
*Oculicosa supermirabilis* venom and fraction #59 modifies hK_v_1.5 currents expressed in MEL cells. **(A)** shows the current traces recorded under normal conditions (black), in the presence of complete venom (10 µg/ml; red), with fraction #59 or Osu1 (6.5 µg/ml, ∼0.9 µM; blue) and recombinant rOsu1 (3 µM; green). The voltage protocol is shown above the figure. The current traces were fitted with a single-exponential function (yellow dashed lines), and the tau values for each trace are indicated with the appropriate color coding. **(B)** displays I-V curves where the ionic currents were recorded under normal conditions and were evoked from −100 mV holding potential to different depolarizing potentials: from −10 mV up to +50 mV. The appropriate membrane potential values are indicated next to the traces along with the tau values coming from a single-exponential fitting.

After an initial screening of some arachnid venoms (data not shown), the venom of *Oculicosa supermirabilis* showed activity over hK_v_1.5. Fractionation of crude venom by reversed-phase HPLC resulted in more than 60 fractions that were manually collected and assayed for biological activity toward hK_v_1.5, mice, and crickets (Figure 1). Although fractions #59, #61 and #67 were toxic to insects, only fraction #59 presented activity affecting the hK_v_1.5 (Figure 2). The before-mentioned fraction was analyzed by mass spectrometry, and it was further purified again by reversed-phase chromatography. As confirmed by analytical chromatography and mass spectrometry, the fraction #59 was obtained at a high purity level and was named Osu1. It represented a concentration of *ca* 60 µg per mg of the dry crude venom of *O. supermirabilis*. The data obtained from automated direct Edman sequencing of the reduced-alkylated fraction #59, and later from endoproteolytic cleavages followed of digested peptide purification, and again N-terminal Edman degradation of such peptide fractions allowed the complete determination of the primary structure of Osu1.

Briefly, direct Edman degradation of the alkylated fraction #59 provided an unambiguous sequence up to amino acid at position 44 (Table 1). Some of the remaining alkylated fraction #59 was enzymatically cleaved by Lys-C, and peptide fractions were collected by RP-HPLC (Supplementary Figure S1). The N-terminal direct sequencing of the alkylated fraction #59 and three of such Lys-C digested peptide fragments allowed the identification of 63 residues of the Osu1 primary structure. Additionally, because of a difference of practically 128 atomic mass units, a Lys residue was placed at position 53 (Table 1, bold), which was also supported by similar amino acid sequence identities found in spider peptide precursors from the venom gland transcriptome of *Lycosa singoriensis* spider (Table 2).

**TABLE 1:** Amino acid sequencing and molecular masses of endoproteolytic fractions from Osu1.

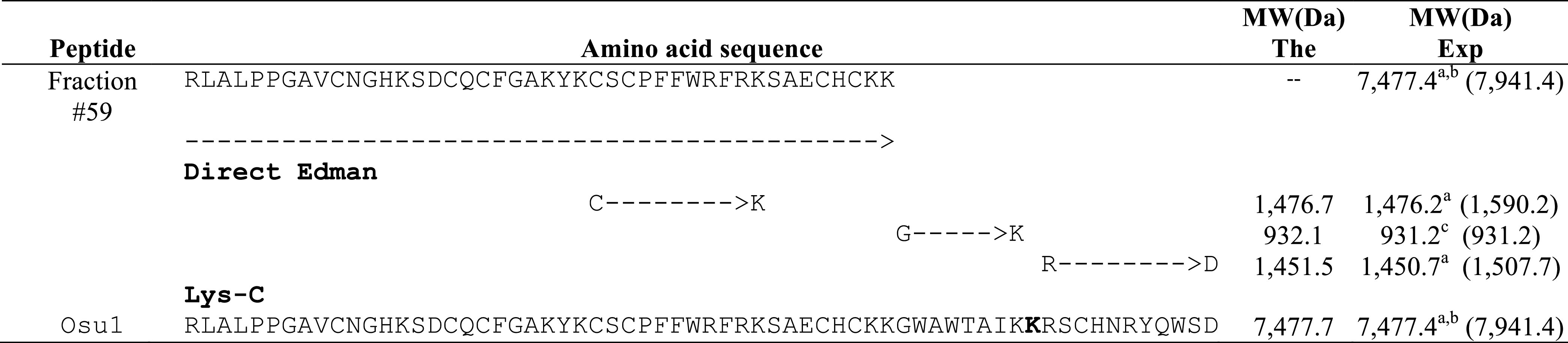

^a^ Molecular mass after subtraction of –57 Da (Cysteine carbamidomethylation by iodoacetamine) to the molecular masses of the alkylated fractions containing Cys (HPLC separation and molecular masses of alkylated fractions are shown in Supplementary Figure S1).

^b^ A subtraction of an extra –8 Da to the alkylated Osu1 was performed assuming four disulfide bridges.

^c^ Non-alkylated peptide fragment. The experimental molecular masses in parenthesis are shown in Supplementary Figure S1.

**TABLE 2:** Alignment of amino acid sequences of Osu1.

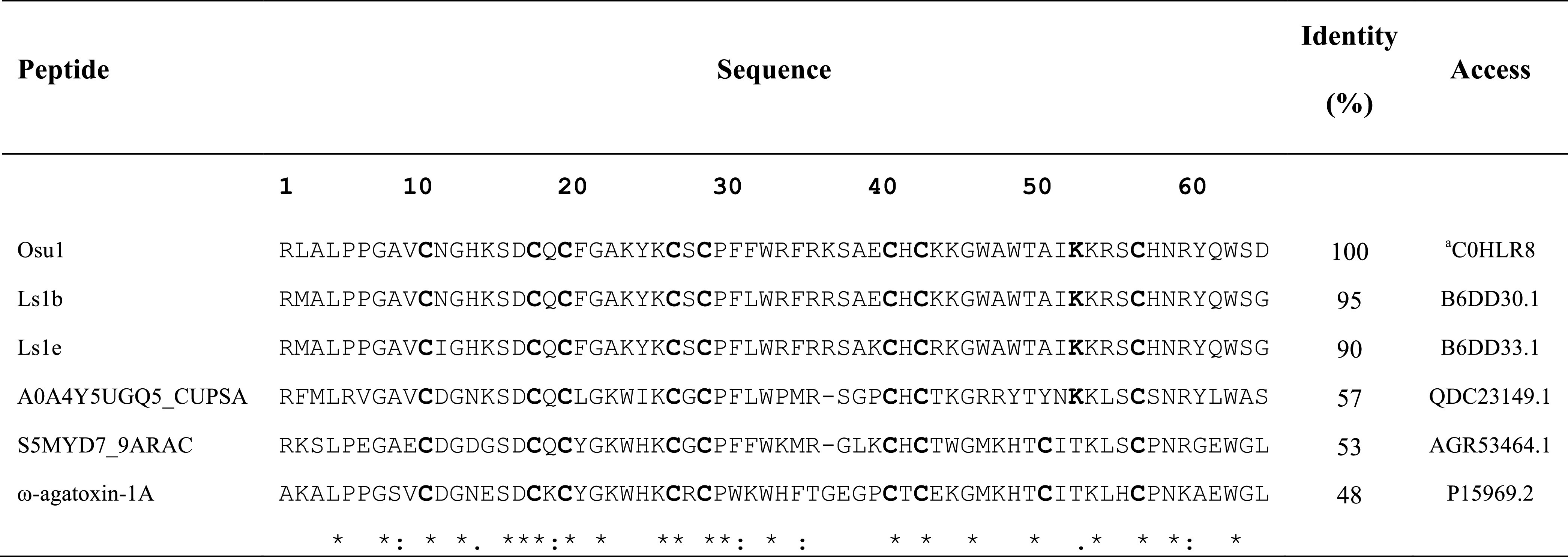

^a^ The protein sequence data reported in this paper will appear in the UniProt Knowledgebase under the accession number C0HLR8. Asterisks represent conserved amino acids. U2-lycotoxin-Ls1b and U2-lycotoxin-Ls1e are from *Lycosa singoriensis;* A0A4Y5UGQ5_CUPSA is from *Cupiennius salei;* S5MYD7_9ARAC is from *Dolomedes mizhoanus;* ω‐agatoxin‐1A is from *Agelenopsis aperta*.

The amino acid sequence agreed on the data gathered from mass spectrometry (Table 1). The estimated theoretical molecular weight of Osu1, assuming pairing the eight cysteine residues into four disulfide bridges, and a free C-terminal carboxylic acid, was 7,477.7 Da. The −0.3 Da mass variation among the calculated and measured molecular mass of Osu1 (7,477.4 Da) may be within the mass spectrometric equipment error. Also, we indirectly speculate that the C-terminal of Osu1 is not amidated based on the transcripts (B6DD30.1 and B6DD33.1) found by Zhang et al. (2010) given that it has not an endoproteolytic amidation signal (Table 2). The amino acid sequence of Osu1 has some identities with toxins from spiders of the same family (Lycosidae), and with others in the same evolutionary clade (Agelenidae and Pisauridae). An automated database search and multiple alignment computations showed that Osu1 had marked identities with peptides from the venom from *Lycosa singoriensis, Cupiennius salei, Dolomedes mizhoanus*, and *Agelenopsis aperta* (Table 2). Most of those toxins have only been registered at the transcriptomic level, and there is no expression evidence within their venom, except for ω-agatoxin-1A, which seems to form heterodimeric structure. However, its three-dimensional structure has not been solved yet (Santos et al., 1992).

### Construction of Osu1

The synthetic oligonucleotides, Osu1-Up1, Osu1-Lw2, Osu1-Up3, and Osu1-Lw4 (Supplementary Table S1), were conveniently assembled using the overlapping oligonucleotide extension, as specified previously in the *Material and Methods* section. The obtained synthetic gene Osu1 was indeed cloned to produce the recombinant plasmid pQE30/Osu1, which was verified by DNA sequencing to confirm the reading frame and the conservation of restriction sites. *E. coli* SHuffle colonies were transfected with the sequenced construct. Some of those colonies could express the peptide Osu1 fused to a His-Tag, as proved by the expression screening of various colonies withholding the plasmid pQE30/Osu1. The expressed protein was confirmed by SDS-PAGE and a band with an apparent molecular weight in the 5–15 kDa region. Then, we picked one of those colonies to overexpress Osu1.

### Recombinant Expression and Purification Osu1 (rOsu1)

**FIGURE 3:**
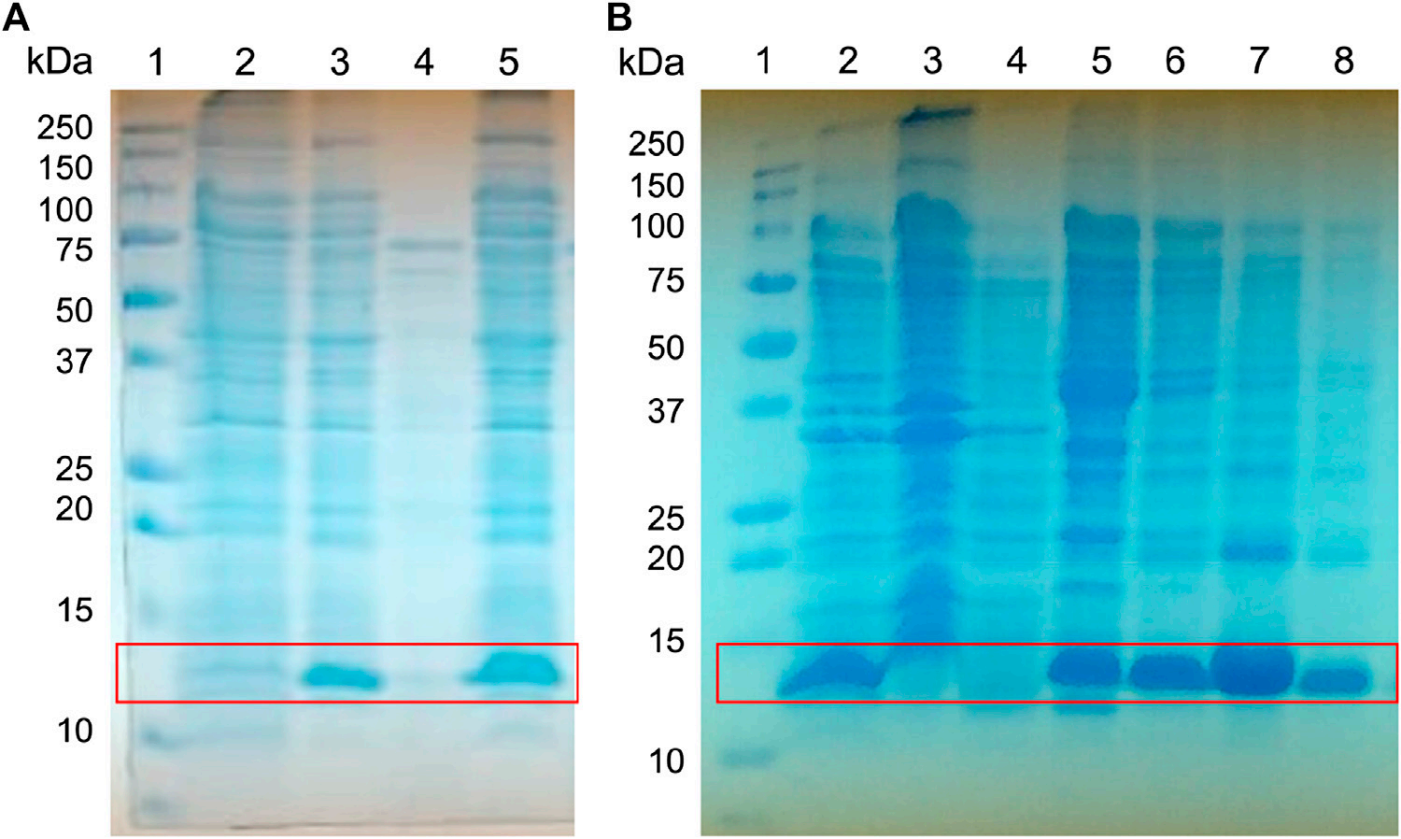
Expression screening of the fusion protein His-Tag-Osu1. Whole-cell lysates were analyzed by reducing **(A)** 15% SDS-PAGE, for the screening of His-Tag-Osu1 expression, identifying the recombinant protein. 1) Molecular weight markers. 2. Non-induced cells. 3. Induced cells. 4. Soluble fraction. 5. Inclusion bodies lysates; and by **(B)** 15% Gel SDS-PAGE, for purification of inclusion bodies using a NiNTA column. 1. Molecular weight marker. 2. Inclusion bodies lysate. 3. Recirculating. 4. First wash with GdHCl 6M, Tris HCl pH 8, 50 mM. 5. Second wash with GdHCl 6 M, Imidazole 40 mM, Tris HCl pH 8, 50 mM. 6–8. Elutions with GdHCl 6M, Imidazole 400 mM, Tris HCl pH 8, 50 mM.

**FIGURE 4:**
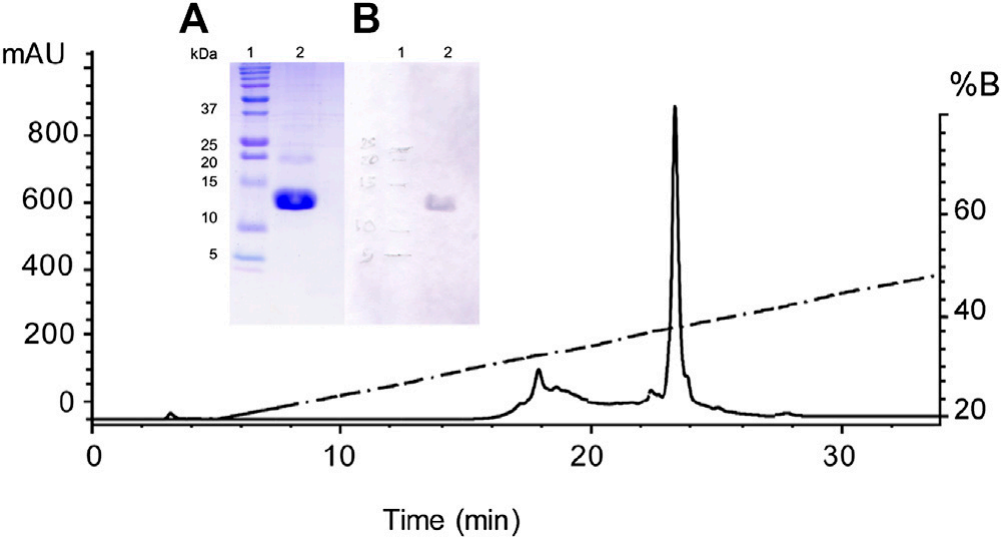
Purification of the recombinant Osu1 by RP-HPLC. Chromatographic separation of the Ni-NTA eluate by RP-HPLC using an analytical C_18_ column and a gradient of aqueous acetonitrile containing 0.1% TFA, starting after 5 min from 20 to 60% CH_3_CN during 40 min at a flow rate of 1 ml/min. Inside figure; **(A)** A 15% SDS-PAGE showing in lane 1 the molecular weight markers in kDa, and in lane 2, the pure recombinant Osu1 from the HPLC chromatogram obtained at the retention time of 23.5 min; and **(B)** A Western-blot showing in lane 1 the molecular weight markers in kDa, and in lane 2, the pure recombinant Osu1 from the HPLC chromatogram obtained at the retention time of 23.5 min developed using an anti-His antibody.

The rOsu1 includes an extra N-terminal sequence of 16 amino acids (MRSGHHHHHHGSIEGR) plus the following Osu1 mature peptide (Table 1). The rOsu1 was expressed using the *E. coli* Shuffle strain (Figures 3A). The expressed proteins were found in inclusion bodies, and they were dissolved utilizing chaotropic agents and purified employing nickel affinity chromatography (NiNTA). SDS-PAGE confirmed the existence of rOsu1; that is, the rOsu1 band was observed between the molecular weight markers of 10 and 15 kDa, which were also observed after the purification of inclusion bodies using the NiNTA column (Figures 3B). The rOsu1 position above 10 kDa under SDS-PAGE obeys mainly to its charge:mass ratio, this unpredictive position under SDS-PAGE has been observed in other recombinant peptides with a high content of basic amino acids (Estrada et al., 2007; de la Rosa et al., 2018). For folding, the cystines of rOsu1 were reduced with DTT and folded *in vitro* in the presence of the GSH/GSSG par redox. After the *in vitro* folding and HPLC purification (Figure 4), the experimental molecular masses of rOsu1 was obtained (9,331.6 Da), and it was in good agreement according to their expected theoretical molecular mass (9,331.7 Da). The expression yield of rOsu1 was calculated *ca* of 0.4 mg of folded peptide/L. The extra N-terminal poly His-tag sequence in the rOsu1 could not be removed because peptide degradation was observed due to non-specific cleavage by FXa (Supplementary Figure S2). Even though the difference between the chromatographic retention time of rOsu1 and the native Osu1, was 0.7 min under similar reverse-phase chromatographic conditions. That is, the chromatographic retention time of rOsu is shorter than the native Osu1because the poly His-tag in rOsu1 makes it a little bit more hydrophilic than its native counterpart (Supplementary Figure S3). Similar retention time differences have been observed between recombinant peptides and native ones (Estrada et al., 2007).

### *In vivo* Biological Activity

The PD_50_ and LD_50_ in house-crickets were calculated using the folded rOsu1 peptide (Table 3). For house crickets, the PD_50_ and LD_50_ of rOsu1 decreased 5.4 and less than 5.3-fold, respectively, compared to the native peptide. Presumably, the extra N-terminal residues of rOsu1 could explain the difference between PD_50_ and LD_50_, compared to the native toxin. These N-terminal residues may interfere in the *in vivo* activity (Estrada et al., 2007). Also, some incorrectly folded rOsu1 may hamper the insecticidal activity compared to the correctly folded rOsu1. However, the rOsu1 has a similar biological effect as the native peptide, indicating a substantial proportion of the recombinant peptide’s correct folding. The biological activity toward insects was similar to other spider peptide toxins (Pineda et al., 2018).

**TABLE 3:** Paralytic and lethal activity of Osu1 and rOsu1.

Peptide	PD_50_ crickets		LD_50_ crickets	
	(µg/g)	(pmol/g)	(µg/g)	(pmol/g)
Control (H_2_0)	—		—	
Native Osu1	0.05 (0.01–0.08)	6.6 (1.3–10.6)	<0.1	<13.3
rOsu1	0.27 (0.23–0.27)	36.1 (30.7–36.1)	0.53 (0.49–0.54)	70.8 (65.5–72.2)

95% confidence intervals (CI) are shown in parentheses.

### Electrophysiology

We tested 30 different animal venoms on the hK_v_1.5 ion channel (see Supplementary Table S2). Five of these showed an effect on the ion current flowing through hK_v_1.5. Of these five, one, the venom of *O. supermirabilis* was further investigated, and the peptide responsible for the effect was determined. First, the whole venom of *O. supermirabilis* was tested on MEL cells, stably expressing hK_v_1.5 channels. Panel A of Figure 2 shows the current traces recorded on MEL cells under control conditions, in the absence of the venom (black), and in the presence of complete venom (10 µg/ml; red). Besides decreasing the current amplitude, we noticed that the kinetics of the ionic current changed and slowed down, indicating that this was not a simple pore inhibition. It is more likely that one of the venom’s peptides is bound to the voltage sensor of the hK_v_1.5 ion channel, thereby altering the gating kinetics of the channel. Therefore, the activation kinetics of ion currents in the presence and absence of venom were determined. Using a single exponential function, we fitted the rising part of the current, and the tau parameter of the fitting proved that the venom’s presence slows down the current activation kinetics. After fractionation of the venom, the effect of each fraction on the hK_v_1.5 currents, was tested. Only one fraction showed an effect, the #59, which we named Osu1. We performed the same experiment with Osu1 (6.5 µg/ml, ∼0.9 µM) as we did with the whole venom described above. Panel A of Figure 2 shows the results with blue: slower activation kinetics, similarly to the venom-experiment. We also tested the recombinant rOsu1 on hK_v_1.5 currents at a concentration of 3 µM, which gave a very similar result to the native Osu1 (see the current trace in Figure 2 in green). The average tau parameters in the presence, and the absence, of rOsu1 were 2.02 ± 0.08 ms and 0.98 ± 0.04 (*n* = 3), respectively. Comparing them with a paired t-test, the difference is significant (*p* = 0.014), indicating that rOsu1 slows down the hK_v_1.5 current activation kinetics significantly. Based on our measurements, rOsu1 binds to hK_v_1.5 in a non-reversible manner (Supplementary Figure S4). Panel B of Figure 2 displays part of the I-V curve. The ionic currents were measured under controlled conditions and were elicited from −100 mV holding potential to different depolarizing potentials: from −10 mV up to +50 mV. The appropriate membrane potential values were indicated next to the traces and the tau values coming from a single-exponential fitting. Panel B also compares the tau values seen in panel A measured in the venom’s presence of Osu1 (natural or recombinant) with the tau values measured under different membrane potentials. So, the tau values obtained in panel A were compared to those of panel B; in this way, we observe some of the effects caused by venom and Osu1 (natural or recombinant). We can conclude that the whole venom caused a shift of about 20–30 mV, while Osu1 (both natural and synthetic/recombinant) affected a variation roughly 30–40 mV.

### Secondary Structure and Proposed Structural Model

**FIGURE 5:**
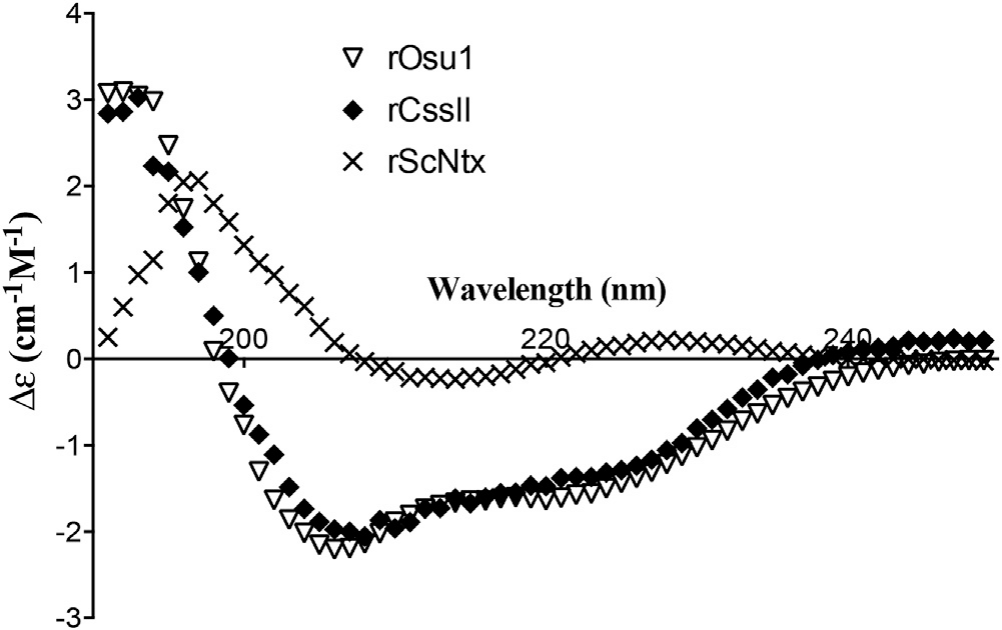
Circular dichroism of recombinant neurotoxins. rOsu1, a recombinant neurotoxin from the venom of the spider *Oculicosa supermirabilis* (This work). rCssII, a recombinant neurotoxin from the venom of the scorpion *Centruroides suffusus suffusus* (Estrada et al., 2007). ScNtx a consensus short “three-finger” recombinant neurotoxin from elapid venom (de la Rosa et al., 2018).

The CD values for the rOsu1 had a minimum and maximum spectrum around 206 and 192 nm, respectively, which represents antiparallel β-sheets (Little et al., 1998). Additionally, α-helix structures were also apparent by another observed minimum at 220 nm (Figure 5). The CD spectrum was evaluated using the deconvolution software from Bestsel (Micsonai et al., 2018), in order to predict the percentages of the secondary structure of rOsu1. The predicted values were 13.6, 23.9, 16.1, 46.4% of α-helix, β-antiparallel, β-turns and random coil, respectively. Taking in account such data, and because the comparison with native Osu1 was not possible because of the low amounts remaining from the crude venom, we decide to compare the rOsu1 secondary structure with that of an α/β motif, which is represented by rCssII, a recombinant neurotoxin from the venom of the scorpion *Centruroides suffusus suffusus* (Estrada et al., 2007; Saucedo et al., 2012). The rCssII also contains an N-terminal poly His-tag, four disulfide bridges, representing a structure with α-helix and β-antiparallel secondary structures. The rCssII had similar CD spectrum as rOsu1; so rOsu1 most likely contain both secondary structures (α-helix and β-antiparallel). Moreover, the CD spectrum of rOsu1 was compared to the CD spectrum of a short “three-finger” recombinant (also with poly His-tag) neurotoxin named ScNtx. Short three-finger toxins are from elapid venoms, and they also contain four disulfides bridged peptides, but its secondary structure is mainly antiparallel (de la Rosa et al., 2018). The CD spectrum of the short “three-finger” neurotoxin differs from that of the rOsu1 and rCssII, suggesting that indeed the secondary structure of rOsu1 and rCssII are not completely β-antiparallel.

**FIGURE 6:**
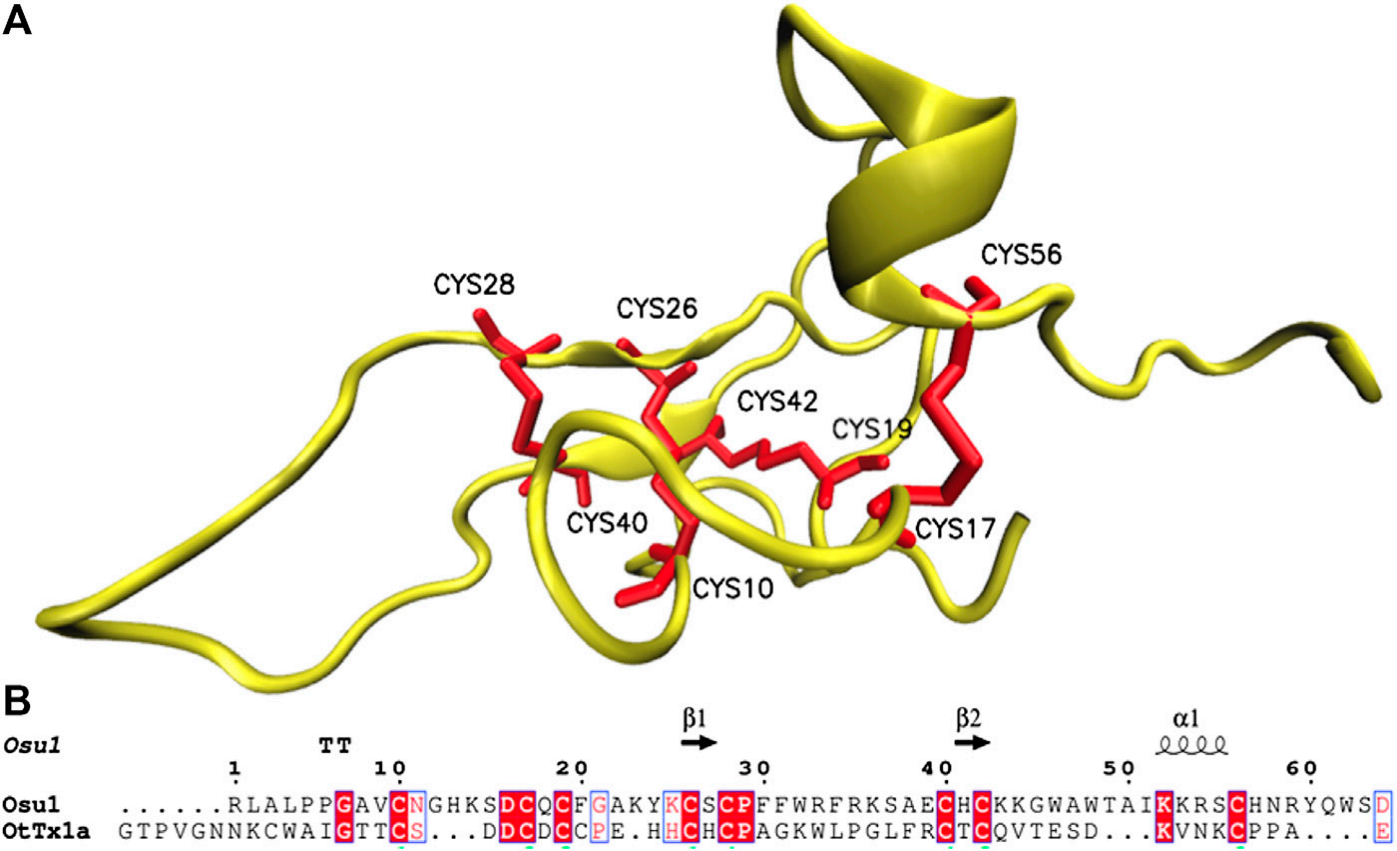
Structural model of Osu1. The structure was generated using Modeller, based on the solved structure of OtTx1a (PDB: 2n86), and it presents a disulfide pairing of Cys28-Cys40, Cys10-Cys26, Cys19-Cys42, and Cys17-Cys56. The protein alignment between Osu1 and OtTx1a showing residues involve in secondary structure is shown below (T means β-turns and the arrows correspond to the antiparallel β-structure).

**FIGURE 7:**
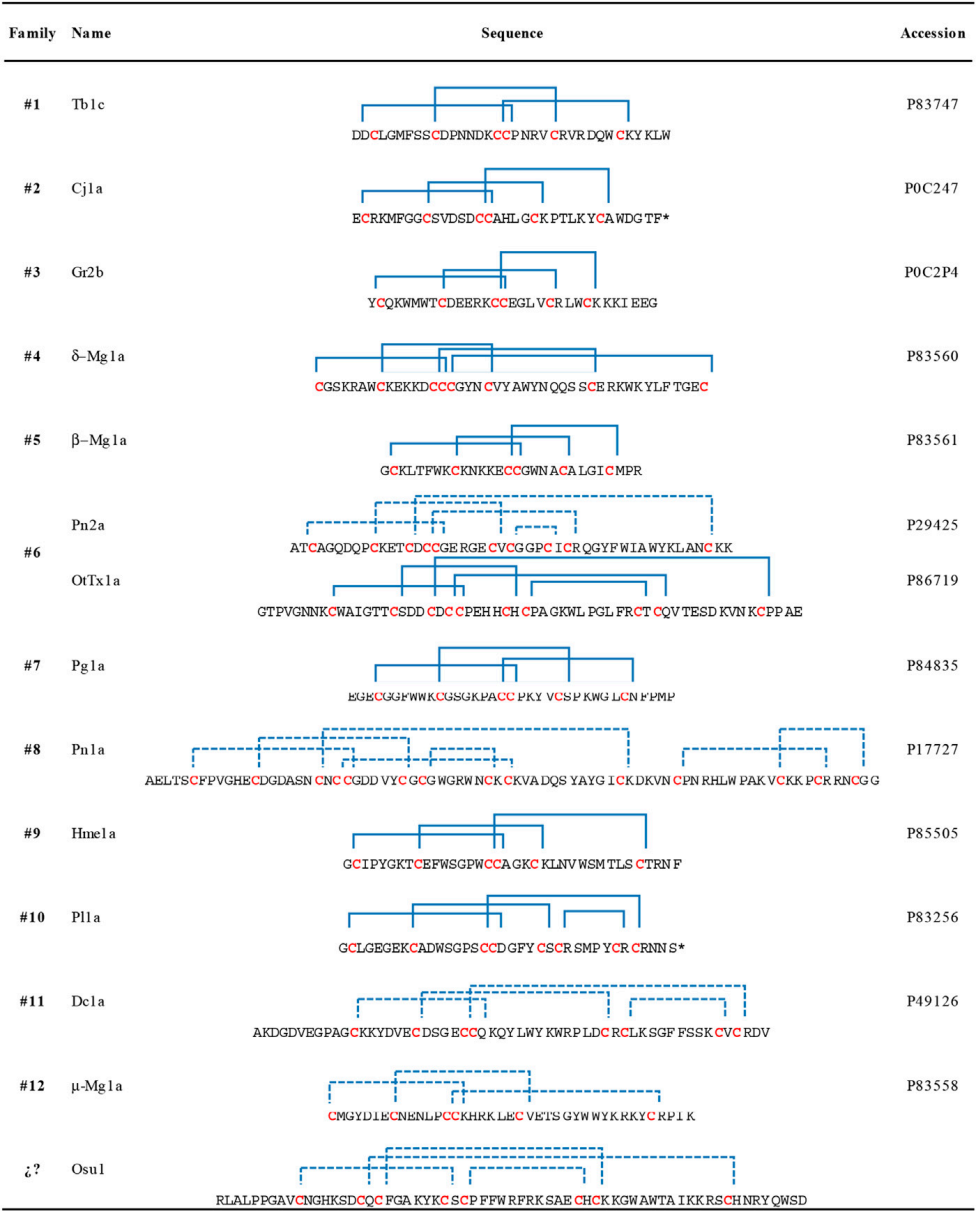
Amino acid sequences and disulfide pairing of a representative member of the proposed families of spider sodium channel toxins, according to Klint et al. (2012) including the proposed primary structure and possible disulfide pairing in Osu1 as a new member of spider toxins. A primary structure of a representative member of each family is shown. Disulfide bridges are colored blue, and blue dotted lines represent predicted disulfide bond connectivities that have not been experimentally validated. Asterisks at the C-terminal mean C-amidation. Here our intention is not to propose Osu1 as new Na_v_ spider toxin, but to use the Klint *et al.* spiders’ toxin classification to show the novelty of the amino acid sequence between Cys residues, and the possible disulfide-pairing motif of Osu1. Toxin names are based on the rational nomenclature devised for spider-venom peptides (King et al., 2008).

Four *in silico* models were created to propose a three-dimensional structure of Osu1 (Supplementary Figure S5). Interestingly, three out of the four protein model programs used (I-Tasser, Modeller, and Swiss-Model) gave similar disulfide bond patterns (residues Cys28-Cys40, Cys10-Cys26, Cys19-Cys42, and Cys17-Cys56). The modeling of proteins located in the “twilight zone” (20–35% protein identity, according to Doolittle, 1986), such as Osu1 concerning OtTx1a (27%), is considered a difficult problem to solve (Doolittle, 1986; Peng and Xu, 2010). Even though, the correct oxidation of all Cys, the same disulfide bond arrangement, and the similar 3D structure (double antiparallel strand) presented by those three modeling programs allow us to propose a 3D structure for Osu1. Interestingly, even though the structure of OtTx1a, used as a template, has an ICK-type disulfide bridge arrangement, the *in silico* Osu1 structure generated with such protein structure programs did not inherit the three-dimensional arrangements of the template. Also, three models (Modeller, Swiss-Model and Robetta) predicted an β-antiparallel and an α-helix secondary structures. According to the CD spectrum of rOsu1, a small α-helix of 10–11 residues may be formed; that is 13.6% of α-helix in the 80 residues of rOsu1. The three models predict an α-helix between positions Cys42 to Cys56. From such three rOsu1 models, the one created by Modeller (Figure 6) was selected to represent the possible structure of Osu1. That model harmonizes with the CD spectrum, has the lowest RMSD value (3.8 Å) of the main chain (N, C, CA, and O). It holds the best percentages of structural quality for the phi and psi angles of 90.9% in the most favorable regions of the Ramachandran plot, giving confidence to such *in silico* model when compared to the other Osu1 models created by I-Tasser or Swiss-Model regarding the template OtTx1a (see Supplementary Table S3 and Supplementary Figure S6). Since the structure created with Modeller, as well as the structures generated with I-Tasser and Swiss-Model, presented the disulfide arrangement Cys28-Cys40, Cys10-Cys26, Cys19-Cys42, and Cys17-Cys56 (Figure 6, top), which is not common in spider peptides motifs (Klint et al., 2012; Langenegger et al., 2019). The mentioned results allow us to speculate that Osu1 represents a new family of spider toxins (Figure 7). According to Klint et al. (2012), the disulfide pairing of OtTx1a seems to belong to the NaSpTx family #6 (Figure 7), but Osu1 certainly did not fix in the same spider toxin family. Here our intention is not to propose Osu1 as new Na_v_ spider toxin, but to use the Klint et al*.* spiders’ toxin classification to show the novel amino acid sequence between Cys residues, and the possible disulfide-pairing motif of Osu1 (Figure 7). That is, Klint et al*.* (2012) classified most of the spider amino acid sequences and possible disulfide bridges for Na_v_ spider peptide toxins. In addition, in their classification, they also included specific spider peptide sequences for K_v_, Ca_v_, and TRP ion channels as well as spider peptide sequences without specific targets. Osu1 does not fix in any of the spider toxin families proposed. So, the speculation that Osu1 may represent a new family of spider peptide toxins based on its possible disulfide bond motif (based on bioinformatics model), and three-dimensional structure must be confirmed by experimental techniques such as NMR or X-Ray crystallography.

## Discussion

To our knowledge, this is the first study examining the venom of the spider *Oculicosa supermirabilis*. A database search was performed to find out toxins of peptide nature similar to Osu1. So, transcriptomic and proteomic reports of spiders belonging to the phylogenetically distant families, such as Sparassidae, Theraphosidae, Viridasiidae, and Theridiidae, have described spider peptides precursors with some degree of identity to Osu1. However, still, none have been detected in such spider venoms by mass spectrometry or N-terminal peptide sequencing (Oldrati et al., 2017). Nonetheless, only peptide toxins with high identities (90–95%) to Osu1 were found in the spider gland transcriptomes, mainly from the Lycosidae and Pisauridae families (Zhang et al., 2010; Jiang et al., 2013). According to this, *O. supermirabilis* belongs to the Lycosidae family but has been phylogenetically related to the Pisauridae family (Zhang et al., 2010; Jiang et al., 2013).

Besides the novelty of its primary structure, Osu1 can modify the ion currents of the hK_v_1.5 in contrast to other K_v_ peptide inhibitors. To our best knowledge, Osu1 is the first peptide that modifies and inhibits the ion current flowing through hK_v_1.5 at a given membrane potential. Based on our results, Osu1 does not bind to the hK_v_1.5 pore, but most likely, it adheres to the VSD of the hK_v_1.5. Consequently, Osu1 could not be considered a pore blocker, since it prevents hK_v_1.5 from opening at given membrane potential. Because of the limited amounts of natural and rOsu1 peptide to conduct the electrophysiological assays, we performed the measurements in a limited way. So, we could not determine a dose-response curve or selectivity measurements over other ion channels.

On the other hand, from the insecticidal perspective, the Osu1 showed paralysis/death effects on crickets, and phenotypically, it induced an excitatory slow-onset impact on them, leading to irreversible spastic paralysis. According to Johnson et al. (1998), this type of effect suggests that the molecular target is likely to be an ion channel found in the CNS. The insecticidal activity of Osu1 is in the range of some spider neurotoxins that affect insects’ Na_v_ channels, such as μ-agatoxin-Aa1a from the spider *Agelenopsis aperta* (LD_50_ of 75.0 pmol/g over *Musca domestica*) (Skinner et al., 1989) and μ-diguetoxin-Dc1a from the spider *Diguetia canities* (PD_50_ 231.0 pmol/g and LD_50_ 13.0 pmol/g, over *Lucilia cuprina*) (Bende et al., 2014). Additionally, the insecticidal activity of Osu1 is in the range of some spider neurotoxins that affect insects’ Ca_v_ channels, such as ω-hexatoxin-Hv1e from the spider *Hadronyche versuta* (LD_50_ of 103 pmol/g over *Acheta domesticus*) (Wang et al., 2001). In contrast to its insecticidal activity, Osu1 was not active in mice when injected intracranially at 0.5 µg/g or 66 pmol/g mouse, compared to spider peptides toxic to mammals such κ-theraphotoxin-Hs1a, a non-selective K_v_ toxin, from the spider *Haplopelma schmidti*, which has an LD_50_ of 0.25 µg/g or 41.5 pmol/g *Mus musculus* after intracranial injection (Liang, 2004). Moreover, the Osu1 activity is not significant compared to the toxic μ-ctenitoxin-Pn1a from the spider *Phoneutria nigriventer*, which has an LD_50_ of 0.047 µg/g or 5.5 pmol/g *Mus musculus* after intracerebroventricular injection (Diniz et al., 1990). Since Osu1 also showed a modulating effect on the hK_v_1.5 channel’s opening, this peptide toxin could be considered as promiscuous, like many other spider toxins with dual or multiple activities toward different receptors. That is, although potassium channels are common targets for various animal peptide toxins, few of such peptide toxins are also paralytic/lethal to insects affecting Na_v_ and/or Ca_v_ ion channels.

Concerning the structure of Osu1, although it is deserving of remembering that any molecular model is uncertain, structural model algorithms have undergone significant development in the last few years to the point that they can predict full structures based just on primary structures. So, the proposed cysteine pattern here (Cys28-Cys40, Cys10-Cys26, Cys19-Cys42, and Cys17-Cys56) in Osu1 does not belong to any of the known disulfide arrangements in spider toxins, such as the inhibitor cystine knot (ICK), disulfide-directed β-hairpin motif (DDH), Kunitz-type, Colipase or MIT1-like, or helical arthropod-neuropeptide-derived motifs (Langenegger et al., 2019). Remarkably, the cysteine arrangement without two cysteine residues in a row is also quite uncommon for large spiders’ peptide toxins. Only in few spider venoms have been reported, such as the ω-agatoxin-IA, a heterodimeric protein with five disulfide bridges which inhibits insect voltage-sensitive Ca^2+^ channels (Pocock and Nicholls, 1992). The sequence identity of ω-agatoxin-IA compared to Osu1 is just 48%. Indeed, the position of cysteine residues in Osu1 resembles in some way to the insecticidal spider toxins from theraphosids such as *Brachypelma, Lasiodora, Grammostola, Chilobrachys, Aphonopelma*, and *Haplopelma* among others, which disulfide connections are considered as disulfide-directed β-hairpin (DDH) motifs. However, such spider toxins are far shorter (*ca* 40 mer) and contain only three disulfide bridges.

Accordingly, Osu1 may be considered a member of a new spider peptide family since its primary structure (XnCX6CX1CX6CX1CX11CX1CX13CXn) and disulfide bridge patterns seem to differ significantly from those primary structures of the families previously proposed (Klint et al., 2012) (Figure 7).

## Concluding Remarks

Osu1 seems to represent a new type of spider toxins based on its primary structures, possible cysteine-bond motif, and modulating effect on hK_v_1.5. Osu1 is insecticidal and might be considered non-toxic to vertebrates. Given that Osu1 can be synthesized by recombinant means and folded correctly, this peptide could be crucial for studying AF and its implications in heart failure, strokes, and other heart-related complications.

## Data Availability Statement

The protein sequence data reported can be found in the UniProt Knowledgebase under the accession number C0HLR8.

## Ethics Statement

All animal experiments were previously approved by the Bioethics Committee of the Biotechnology Institute (project No. A1-S-8005) and carried out following appropriate regulations.

## Author Contributions

SC-A and IA performed the native Osu1 purification; TO-P, FZ, and GC elucidated the primary structure of Osu1; LC-G designed and cloned the Osu1; DA, HC, and LC-G expressed the Osu1; DA and HC performed the biological activity in crickets and mice; FP, AC, and JB performed the electrophysiological experiments; DA and PM-G performed the Osu1 structural models; FP, GP, and GC financed, reviewed and wrote the manuscript.

## Funding

This work received funding from the Dirección General de Asuntos del Personal Académico (DGAPA-UNAM) grant number IN203118 awarded to GC, and from FORDECYT “Venenos y Antivenenos” grant number 303045. The following grants also supported OTKA Bridging Fund 1G3DBKB0BFPF247 (FP); OTKA K119417 (GP); EFOP-3.6.1-16-2016-00022, Ministry of Human Capacities, Hungary (GP); GINOP-2.3.2-15-2016- 00015 (GP). DA and SC-A are MSc students from Programa de Maestría en Ciencias Bioquímicas. PM-D and JB are PhD students from Programa de Doctorado en Ciencias Bioquímicas and Ciencias Biomédicas, both at the Universidad Nacional Autónoma de México (UNAM). All four postgraduate students are supported by CONACyT-México (Fellowship CVUs No. 925354, No. 884453, No. 775900 and No. 487264/415092, respectively).

## Conflict of Interest

The authors declare that the research was conducted in the absence of any commercial or financial relationships that could be construed as a potential conflict of interest.

The reviewer EU declared a past co-authorship with one of the authors GP to the handling editor.
