# Supplementary material for: A Novel Insecticidal Spider Peptide that Affects the Mammalian Voltage-Gated Ion Channel hKv1.5
Source: Front Pharmacol. 2021 Jan 13;11:563858. doi: 10.3389/fphar.2020.563858 (PMC7883638; doi:10.3389/fphar.2020.563858)
Supplement: Supplementary file 1 [file datasheet1.pdf]

Supplemental material

Table S1. Oligonucleotides used for Osu1 gene synthesis

Name	Sequence (5’ – 3’)
Osu1 -Up1	GAGGGATCCATCGAGGGACGCCGCCTGGCGCTTCCGCCTGGCGCCGT GTGCAACGGTCATAAGAGTG
Osu1 -Lw2	CCAAAAGAACGGGCAGCTACACTTATATTTCGCGCCAAAGCACTGAC AATCACTCTTATGACCGTTGCAC
Osu1 -Up3	GCTGCCCGTTCTTTTGGCGTTTCCGCAAAAGTGCAGAATGTCACTGCA AGAAAGGTTGGGCGTGGACC
Osu1 -Lw4	CTCCTGCAGCTATTAGTCACTCCATTGGTAGCGATTATGACAGCTACG TTTCTTAATGGCGGTCCACGCCCAACC

Table S2. Animal venoms tested on hK_v1.5.

Scorpions	Spiders	Others
Androctonus crassicauda	*Aphonopelma serratum*	*Paragaleodes pallidus*
Centruroides tecomanus	*Brachypelma albiceps*	*Ambrysus lunatus*
Centruroides suffusus suffusus	*Brachypelma auratum*	
Centruroides noxius	*Brachypelma smithi*	
Centruroides limpidus	*Dolomedes gertschi*	
Centruroides gracilis	*Drassodes sp.*	
Hadrurus gertschi	*Dysdera sp.*	
Hadrurus sp	*Eresus sp.*	
	Gnaphosa taurica	
	Lachesana sp.	
	Misumena vatia	
	Oculicosa supermirabilis	
	Palpimanus sp.	
	Phidippus octopunctatus	
	Phyxioshema sp.	
	Pisaura mirabilis	
	Sparassus sp.	
	Steatoda grossa	
	Thomisus onustus	
	Tibellus oblongus	

Table S3. Comparative table that show statistical parameters of different Osu1 models obtained from 3D prediction software

	**I-Tasser**	**Modeller**	**Swiss-model**	**Robetta**
Method	Threading	Homology	Homology	Ab-initio
Cys-Cys bonds	Cys10-Cys26<br>Cys17-Cys56<br>Cys19-Cys42<br>Cys28-Cys40	Cys10-Cys26<br>Cys17-Cys56<br>Cys19-Cys42<br>Cys28-Cys40	Cys10-Cys26<br>Cys17-Cys56<br>Cys19-Cys42<br>Cys28-Cys40	Cys10-Cys42<br>Cys17-Cys28<br>Cys19-Cys56<br>Cys26-Cys40
RMSD	5.3	3.8	4.4	11.3
Ramachandran plot MFR (%)	36.4	90.9	63.3	89.1
Ramachandran plot AAR (%)	52.7	5.5	34.7	9.1
Ramachandran plot GAR (%)	9.1	1.8	2	0
Ramachandran plot DR (%)	1.8	1.8	0	1.8
Secondary structure	Antiparallel	Antiparallel and helix	Antiparallel and helix	Antiparallel and helix

MFR=Most favored regions
AAR=Additional allowed regions
GAR=Generously allowed regions
DR= Disallowed regions

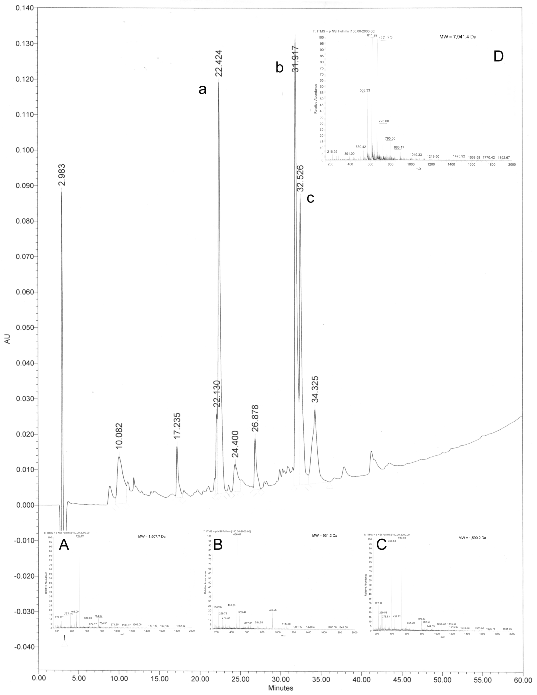

Figure S1. Reverse-phase separation of alkylated fractions. The alkylated native Osu1 was enzymatically cleaved by Lys-C. The chromatographic separation was performed using an analytical C_{18} column and a gradient of aqueous acetonitrile containing 0.1% TFA (solution B), starting after 5 min from 0 to 60% CH_3CN during 60 min at a flow rate of 1 mL/min. Mass spectra in panels A, B and C correspond to HPLC fractions to retention times 22.4, 31.9 and 32.5 min (a, b and c), respectively. Mass spectrum in panel D corresponds to the full alkylated Osu1.

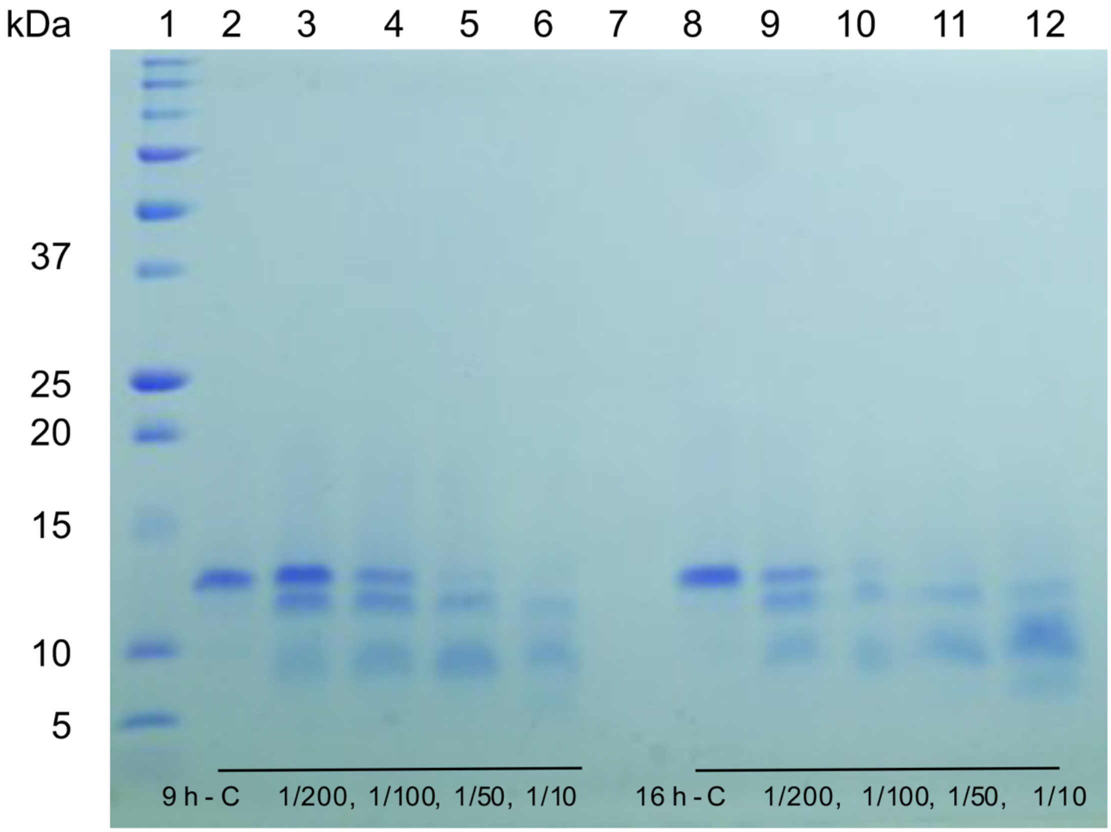

Figure S2. Endoproteolytic cleavage of rOsu1 by Factor Xa. Lane 1, Molecular weight markers; Lane 2, rOsu1 control sample; Lanes 3-6, cleavage for 9 h. Lane 8, rOsu1 control sample; Lanes 9-12, cleavage for 16 h. The Fxa to rOsu1 ratio is shown. 15% SDS-PAGE.

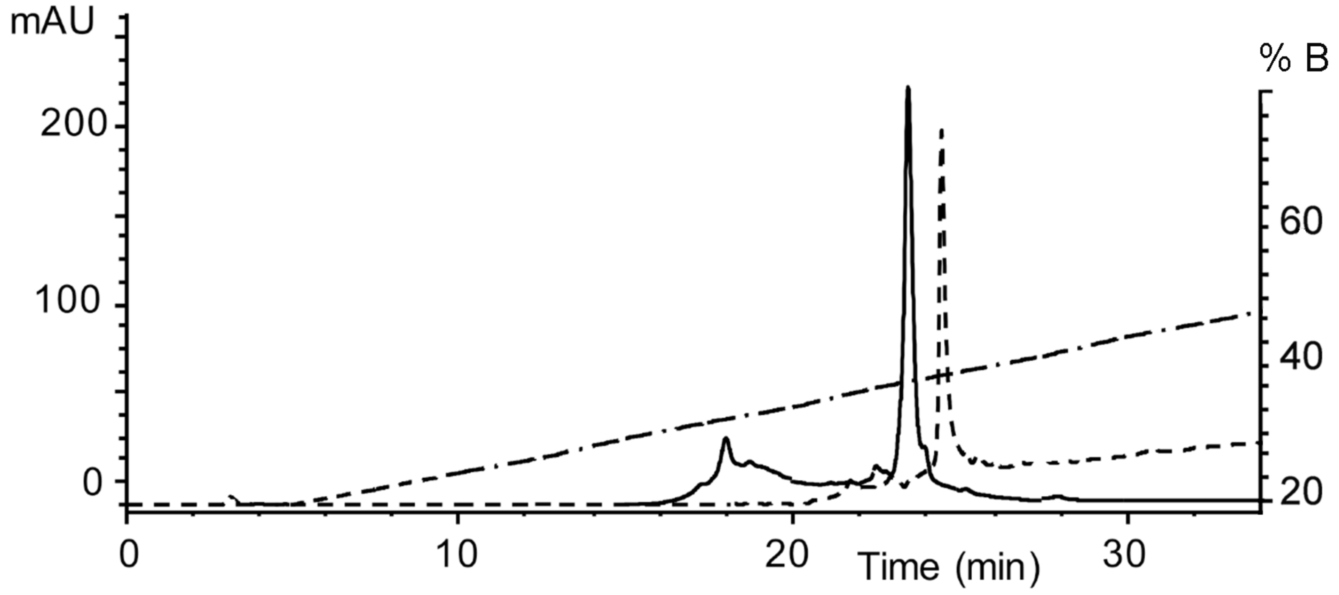

Figure S3. Comparative reverse-phase separation of rOsu1 and native Osu1. Superimposed in this chromatogram is a profile of rOsu1 (solid line) and native Osu1 (dashed line). Chromatographic separation was performed in a RP-HPLC using an analytical C_{18} column and a gradient of aqueous acetonitrile containing 0.1% TFA (solution B), starting after 5 min from 20 to 60% CH_3CN during 40 min at a flow rate of 1 mL/min.

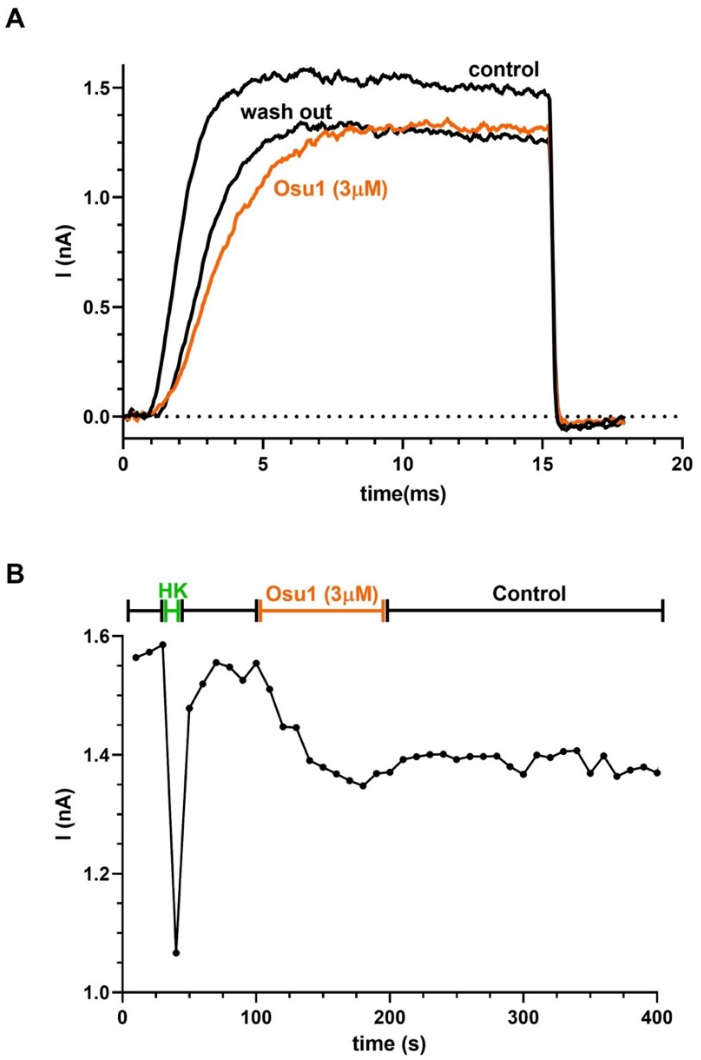

Figure S4. Recombinant *Osu1* effect is not reversible on hK_v1.5. Panel **A** shows the current traces recorded under control conditions (black) and in the presence of recombinant Osu1 (3 µM; orange) as a response to +50 mV depolarization. Panel **B** displays the peak current amplitudes during +50 mV depolarization pulse. Black horizontal bars represent that the peak currents were measured under control conditions; orange bar indicates the presence of Osu1 in the external solution and green bar refers to perfusion with high potassium external solution (HK; 150 mM K^+) to test the perfusion system on this given cell.

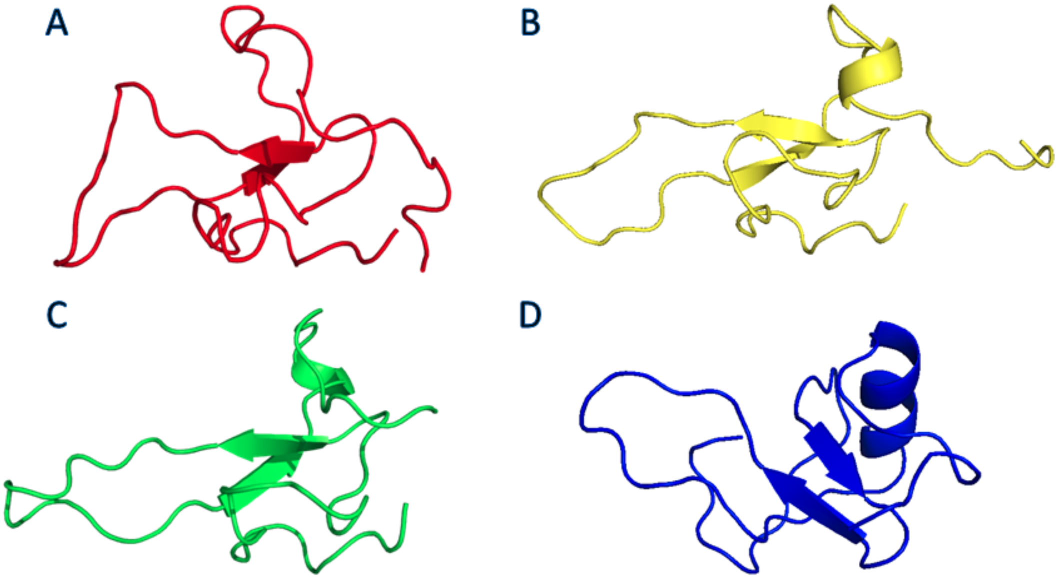

Figura S5. Three-dimensional models of Osu1 obtained from different modeling programs. A) I-Tasser. B) Modeller. C) Swiss-model. D) Robetta.

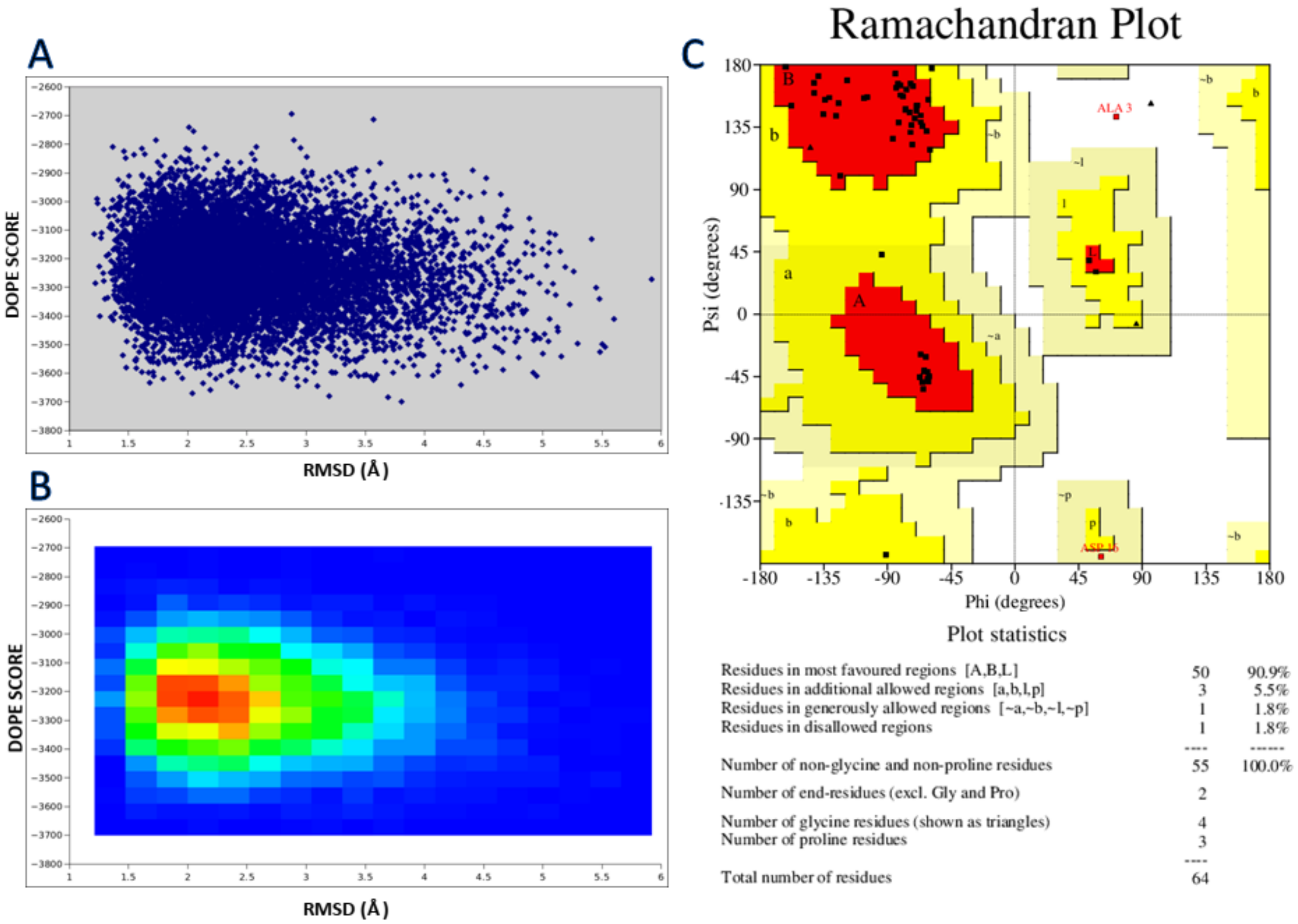

Figure S6. Osu1 models generate by the server Modeller. A) Distribution of 10,000 models generated by Modeller. B) Distribution of density population of 10,000 models generated by Modeller. C) Ramachandran graphics for the best model according to the percentage of residues in the favorable regions.
